# Optimal Methylammounium Chloride Additive for High-Performance Perovskite Solar Cells

**DOI:** 10.3390/nano15040292

**Published:** 2025-02-14

**Authors:** Qinghua Cao, Hui Liu, Jiangping Xing, Bing’e Li, Chuangping Liu, Fobao Xie, Xiaoli Zhang, Weiren Zhao

**Affiliations:** 1 Shanwei-GDUT Collaborative Research Institute for Innovation Industrial Technology, Shanwei 730010, China; 2 School of Physics and Opto-Electronic Engineering, Guangdong Provincial Key Laboratory of Sensing Physics and System Integration Applications, Guangdong University of Technology, Guangzhou 510006, China; 2112415045@mail2.gdut.edu.cn (Q.C.); liuhui@gdut.edu.cn (H.L.); 2112415036@mail2.gdut.edu.cn (J.X.); 2112315061@mail2.gdut.edu.cn (B.L.); 2112315025@mail2.gdut.edu.cn (C.L.); 2112415145@mail2.gdut.edu.cn (F.X.); xlzhang@tju.edu.cn (X.Z.)

**Keywords:** perovskite film quality, MACl additive, high-performance PSCs

## Abstract

Organic–inorganic lead halide perovskite solar cells (PSCs) have presented promising improvements within recent years due to the superior photophysical properties of perovskites. The efficiency of PSCs is closely related to the quality of the of the perovskite film. Additive engineering is an effective strategy to regulate the crystallization of perovskite film. Therefore, in this work, we introduce methylammounium chloride (MACl) into a perovskite precursor as an additive to improve the crystallization of perovskite film and to suppress the formation of defects to achieve high-performance PSCs. By meticulously investigating and studying the influence of different percentages of MACl additives on perovskite film quality, we obtain that the best amount of incorporated MACl is 10%. Thanks the employment of the optimal amount of MACl, the perovskite film shows a significantly improved morphology with larger grains, a smoother surface, and suppressed defects. Finally, the target PSCs with the addition of 10% MACl present the highest PCE of 23.61%, which is much higher than the value (16.72%) of the control device.

## 1. Introduction

Due to the superior photophysical properties of perovskites, such as a high absorption coefficient, high carrier mobility, long carrier lifetime, low exciton binding energy, direct and adjustable bandgap, and so on, organic–inorganic lead halide perovskite solar cells (PSCs) have presented promising improvements over recent years. The power conversion efficiency (PCE) of PSCs has been increased from 3.8% in 2009 to over 26% in 2024, suppressing the efficiency of silicon solar cells which have dominated in the market [1,2,3,4]. The efficiency of PSCs is closely related to the quality of perovskite films. Nowadays, many reports focus on different strategies like solvent regulation, gas–atmosphere regulation, composition engineering, and additive engineering to form high-quality perovskite films with smooth and compact film surfaces, a low roughness, dense and large grains, high crystallinities, and the preferred orientations, as well as low trap defects [5,6,7,8]. In particular, the introduction of additives into perovskite precursors is an effective way to participate in the nucleation of perovskites, and to regulate the crystallization kinetics during the formation of perovskite film.

Among diverse additives, chloride-based additives present significant potential in adjusting the nucleation and growth of perovskite grains, regulating the quality of film morphology, as well as decreasing trap density and suppressing trap-induced non-radiative recombination, helping achieve high a PCE for PSCs [9,10,11]. Henry J. Snaith et al. carried out a comprehensive investigation into the influence of methylammonium chloride (MACl) on a mixed-cation mixed-halide perovskite through studying the formation of an MACl-assisted intermediate phase [12]. They also studied the role of a series of halide salt additives, including formamidinium chloride, cesiumchloride, methylammoniumiodide, and methylammonium bromide, and found that the MACl-based perovskite films showed good abilities to suppress halide segregation and prolong ambient stability. Paul J. Dyson and Mohammad Khaja Nazeeruddin et al. employed [Bcmim]Cl and MACl additives, which interacted to inhibit the condensation reaction between MACl and FAI, leading to a high-quality film with large and oriented grains and an improved phase purity and finally high-efficient and stable perovskite photovoltaics [13]. Therefore, it has been confirmed that the introduction of a MACl additive into perovskites demonstrates strong effects on the growth of high-quality perovskite films. However, different perovskite systems present unique requirements for the optimal amount of MACl additives.

In this work, we introduce MACl into a perovskite precursor as an additive to improve the crystallization of perovskite film and to suppress the formation of defects to achieve high-performance PSCs. By meticulously investigating and studying the influence of different percentages of MACl additives on perovskite film quality, we find that the optimal amount of incorporated MACl is 10%. Thanks to the employment of optimal MACl, the perovskite film shows a significantly improved morphology with larger grains, smoother surface, and suppressed defects. Finally, the target PSCs with the addition of 10% MACl present the highest PCE of 23.61%, which is much higher than the value (16.72%) achieved by the control devices, and also larger than most of the reported studies based on MACl additives.

## 2. Experimental Section

### 2.1. Material

Methylammonium chloride (MACl, 99.99%), lead bromide (PbBr_2_, 99.99%), cesium iodide (CsI, 99.99%), formamidine hydroiodide (FAI, 99.99%), and (6,6)-Phenyl C61 butyric acid methyl ester (PC_61_BM, 99.99%) were purchased from Xi’an Yuri Solar Co., Ltd. Xian, China. Lead iodide (PbI_2_, 99.99%), Methylammonium bromide (MABr, 99.99%), and bromocresol purple (BCP, 99.99%) were purchased from Advanced Election Technology Co., Ltd., Liaoning, China. 2-Phenylethylamine Hydroiodide (PEAI, 99.99%) and methylammonium iodide (MAI, 99.99%) were ordered from Xian Polymer Light Technology Corp. Xian, China. 2-(3,6-Dimethoxy-9H-carbazol-9-yl)ethyl)phosphonic acid (MeO-2PACz, 99.99%) was purchased from Derthon Optoelectronics Materials Science Technology Co., Ltd., Shenzhen, China. N, N-dimethylformamide (DMF, 99.99%), dimethyl sulfoxide (DMSO, 99.99%), isopropanol (IPA), and chlorobenzene (CB, 99.99%) were purchased from Sigma-Aldrich, St. Louis, MO, USA.

### 2.2. Device Fabrication

The FTO substrates were cleaned using DI water, acetone, and IPA in an ultrasound bath for 20 min, respectively. Then, they were treated under UV ozone for 20 min before being transferred to the N_2_ glove box for film formation.

A MeO-2PACz (0.6 mg/mL in ethanol) solution was deposited on the substrate at 3000 rpm for 30 s, followed by annealing at 100 °C for 10 min. Then, the control perovskite precursor solution was prepared with 66 mg PbBr_2_, 20.1 mg MABr, 588.3 mg FAI, 18.2 mg CsI, and 1716 mg PbI_2_ dissolved in 2076 μL DMF and 519 μL DMSO mixed solution. Optimized perovskite precursors were added with different percentages of MACl additives (the MACl percentage is the ratio relative to the molar concentration of the perovskite precursor solution). After stirring for 6 h, perovskite films were formed by spin-coating on MeO-2PACz substrates at 1000 rpm for 5 s and then 4000 rpm for 30 s. Then, 160 μL CB antisolvent was dropped onto the film 15 seconds before the end of the spin-coating process, followed by annealing at 100 °C for 50 min. PEAI and MAI (at a mass ratio of 2:1 in an IPA and DMF solution with a volume ratio of 150:1) were dynamically spin-coated onto the surface of the perovskite film at 5000 rpm for 30 s, followed by annealing at 100 °C for 5 min. Then, PCBM (20 mg/mL in CB) was spin-coated at 1500 rpm for 35 s, and annealed at 100 °C for 10 min. BCP (0.5 mg/mL in IPA) was then dynamically spin-coated at 4000 rpm for 30 s. Finally, Ag (100 nm) was thermally evaporated to form the electrode.

### 2.3. Characterization and Test

The morphologies of the perovskite films were characterized by scanning electron microscopy (SEM, JEOL JSM-7800F Prime, Japan Electronics Co., Ltd., Anjo, Japan). The surface roughness was tested using a MultiMode 8 and Dimension Icon Atomic Force Microscope (AFM, Bruker (Beijing) Technology Co., Ltd., Seoul, Republic of Korea) in tapping amplitude modulation mode. UV-vis spectra were characterized with a UV-vis spectrometer (UV-3600, Weihai Optical Instrument (Shanghai) Co., Ltd., Weihai, China). The XRD patterns were measured using X-ray diffraction (XRD) carried out on a Bruker D2 Phaser with Cu Kα radiation. The steady-state photoluminescence spectrum (PL) and time-resolved photoluminescence spectrum (TRPL) were measured with a fluorescence spectrophotometer (FLS980, Edinburgh Instruments, Edinburgh, UK).

The current density–voltage (J-V) was measured with a Keithley 2400 digital source meter under a simulated solar source of AM1.5 G (100 mW/cm^2^, Wacom Denso Co., Kariya, Japan). A shading mask with an aperture area of 0.04 cm^2^ was used to ensure the accuracy of the current density in the J-V curves.

## 3. Results and Discussion

### 3.1. Optimal Amount of Introduced MACl Additive

**Figure 1:**
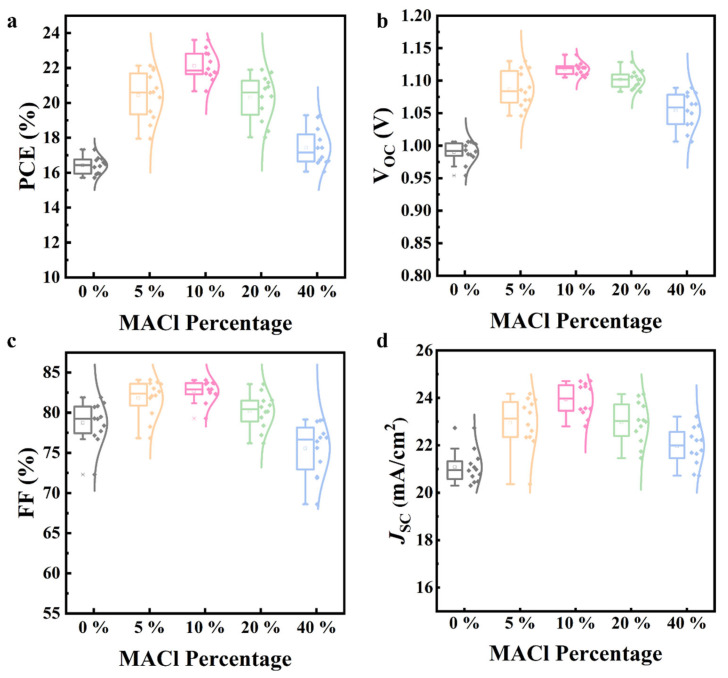
Statistical distribution of (**a**) PCE, (**b**) Voc, (**c**) FF, and (**d**) Jsc of PSCs with different percentages of MACl additives.

In order to obtain the statistical distributions of the photovoltaic parameters of the PSCs, and determine the optimal percentage of MACl additive to introduce to them, we firstly studied the performance of PSC devices with different percentages of MACl by testing 12 devices manufactured using an identical fabrication process. As illustrated in Figure 1 and Table S1 in the Supplementary Materials, all of the parameters of the PSCs gradually increased when the percentage of MACl increased from 0% to 5%, and to 10%, including the open-circuit voltage (Voc), fill factor (FF), and short-circuit current density (Jsc). However, further increasing the amount of MACl additive resulted in a clear drop in Voc, FF, and Jsc, leading to a final drop in the PCE of the PSCs. Therefore, the best percentage of introduced MACl additive was evaluated to be 10% (i.e., the percentage of additive relative to the molar concentration of the perovskite precursor solution), which delivered the highest average Voc (1.11 V), FF (82.66%), Jsc (23.93 mA/cm^2^), and PCE (22.12%). The employment of an optimal amount of MACl additive can significantly contribute to morphological improvements and grain growth as well as a decrease in defects, which leads to a significant enhancement of device performance. Further details will be analyzed in the following section.

### 3.2. Improvement in the Quality of Perovskite Films by Employing MACl

**Figure 2:**
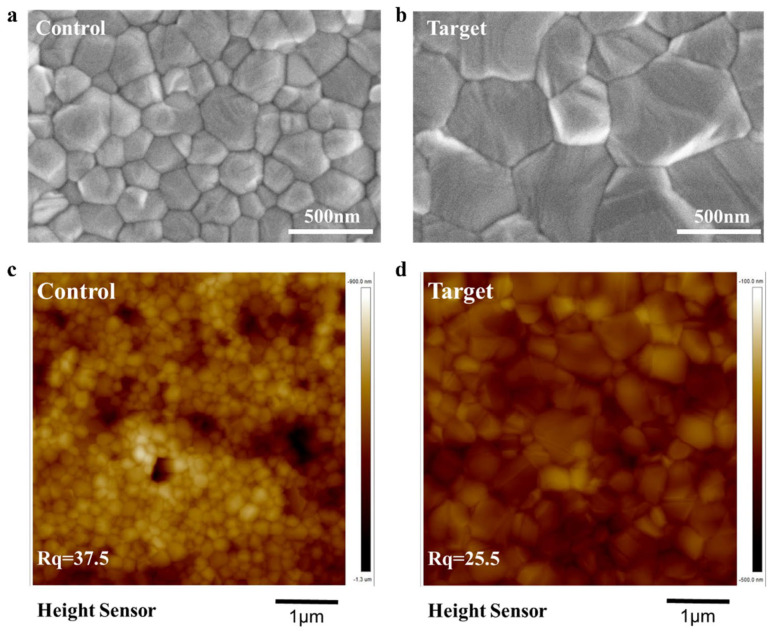
SEM images of (**a**) control perovskite film and (**b**) target perovskite film; AFM images of (**c**) control perovskite film and (**d**) target perovskite film.

We then investigated the influence of introducing the optimal amount of MACl additive on the qualities of the perovskite films. Scanning electron microscopy (SEM) characterization was used to observe the morphology of control perovskite film (without the addition of an MACl additive) and the target perovskite film (including 10% MACl additive), as shown in Figure 2a,b. It was found that the control perovskite film clearly exhibits smaller grain sizes (around 252.36 nm), and the grains show a randomly nonhomogeneous distribution, which indicated the uncontrollable crystallization process of perovskite film formation [14,15]. Surprisingly, following the incorporation of the optimal amount of MACl additive, the target perovskite film’s quality was significantly improved, exhibiting a significant increase in grain size (about 539.05 nm) and a reduced grain boundary, suggesting that the MACl additive assists the recrystallization of perovskites during the Ostwald ripening process [16,17]. With a further increase in the amount of additive, the perovskite films showed larger grain sizes, but the films’ surfaces and compactness became worse. SEM images of perovskite films with 5%, 20%, and 40% additions of MACl and distribution histograms of the grain sizes are shown in Figures S2 and S3 in the Supplementary Materials, respectively.

Moreover, atomic force microscopy (AFM) was conducted to study the surface roughness of the control perovskite film and the target perovskite film. As presented in Figure 2c,d, the control film without any MACl additive displays a rougher surface, with a root mean square (RMS) of 37.5 nm. However, the target perovskite film becomes smoother, and the RMS value largely decreases to 25.5 nm, which is the lowest roughness among the different concentrations of MACl additives (Figure S4 in Supplementary Materials). AFM 3D images of the control and target perovskite films are shown in Figure S1 in the Supplementary Materials. A smooth surface is conducive to forming the desired interface contact with the carrier transport layer, suppressing carrier recombination at the interface, which effectively contributes to the higher Voc of PSCs.

**Figure 3:**
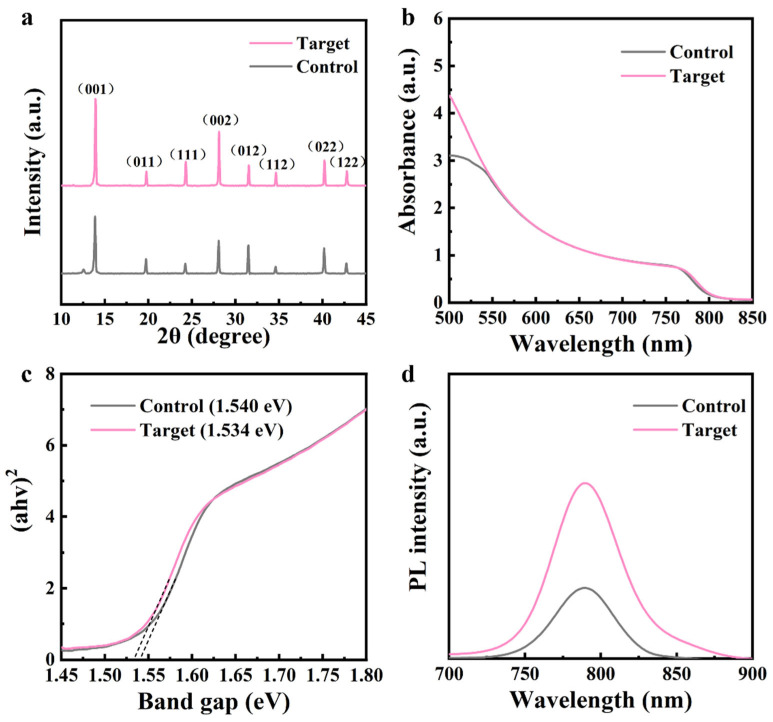
(**a**) XRD spectra, (**b**) absorbance spectra, (**c**) Tauc plots, and (**d**) PL spectra of control and target perovskite films.

Furthermore, the influence of the employment of an MACl additive on the crystallization of perovskites was investigated by X-ray diffraction (XRD). As shown in Figure 3a, the peak positions of the control and target films are identical, which indicates that the crystal phases of perovskite films do not change following the introduction of a MACl additive [18]. However, the crystallinities of target perovskite peaks are significantly increased after the incorporation of MACl, especially the diffraction intensity of the (001) lattice plane of perovskites. Meanwhile, the peak of PbI₂ is almost entirely diminished. The crystallinity of perovskites is gradually enhanced with the increase in the amount of MACl additives (Figure S5 in Supplementary Materials). Therefore, it is clear that MACl additives help improve the crystallization of perovskites and reduce the phase of PbI_2_, which will be beneficial for the enhancement of the performance of PSC devices. The increased light absorption intensity of perovskite film also indicates the enhanced crystallization quality of perovskites. It should be noted that the introduction of the MACl additive does not affect the band gap of the perovskites (Figure 3b,c and Figure S6 in Supplementary Materials). The band gap of the target perovskite film is 1.534 eV, similar to that of the control perovskites (1.540 eV) with negligible change. In addition, the target perovskite film with the introduction of the optimal amount of MACl additive shows the highest PL intensity and the longest lifetime compared to the control film (Figure 3d) and films with other additive concentrations (Figure S7 in Supplementary Materials), which suggests a suppressed trap density and non-radiative recombination [19].

### 3.3. Performance of PSC Devices

**Figure 4:**
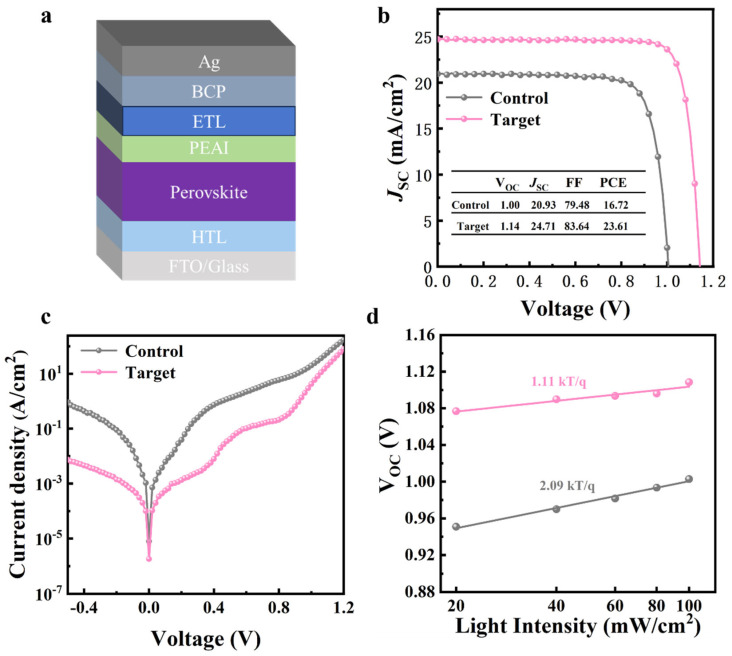
(**a**) Diagram of structure of PSCs; (**b**) J-V curves of best-performing target PSCs fabricated with 10% MACl additive and control device; (**c**) dark current curves of control and target PSCs; (**d**) light intensity dependence of Voc of control and target PSCs.

The inverted PSCs were fabricated with a structure of glass/FTO/HTL (MeO-2PACz)/perovskite/PEAI/ETL (PC_61_BM)/BCP/Ag, as shown in Figure 4a. The target PSCs achieves the highest efficiency. J-V curves of best-performing target PSCs fabricated with 10% MACl additive and control device are presented in Figure 4b. The Voc, Jsc, FF, and PCE values of the target device are 1.14 V, 24.71 mA cm^2^, 83.64%, and 23.61%, respectively, while the values of these metrics for the control PSCs are 1.00 V, 20.93 mA cm^2^, 79.48%, and 16.72%, respectively. J-V curves of PSCs with other concentrations of additives are shown in Figure S8 in the Supplementary Materials. The efficiency of the target PSCs is also higher than that reported in most studies based on MACl additives (Table S2, Supplementary Materials). The J-V characteristics of the control and target devices in the dark conditions were further assessed to compare the dark currents of the PSCs, as shown in Figure 4c. The PSCs with MACl additives presented significantly decreased dark currents, which demonstrated the effectively reduced defects and traps. Finally, the light intensity-dependent Voc was tested, as shown in Figure 4d. It is expressed by the equation of qVoc = E_g_ + mK_B_Tln(I), where q denotes the elementary charge, E_g_ is the band gap energy of perovskites, m stands for the ideality factor, K_B_ refers to the Boltzmann’s constant, T denotes the absolute temperature, and I is the light intensity. The ideality factor approaching 1 indicates decreased non-radiative recombination [20]. The ideality factor of the control PSCs was 2.09 while that of target device decreased to 1.11, implying that defects within the PSCs were effectively suppressed and non-radiative recombination was largely suppressed by the employment of the MACl additive. The target PSCs with optimal MACl additive is also superior to devices with other concentrations of additives (Figure S9 in Supplementary Materials) [9,11,14,16,17,21,22,23,24,25].

## 4. Conclusions

In this work, we introduced the MACl into perovskite precursor as an additive to improve the crystallization of perovskite film and suppress the formations of defects to achieve high-performance PSCs. By meticulously investigating and studying the influence of different percentages of MACl additives on perovskite film quality, we found that the best amount of MACl to incorporate is 10%. Thanks to the employment of the optimal MACl concentration, the perovskite film shows a significantly improved morphology with larger grains, a smoother surface, and suppressed defects. Finally, the target PSCs with the addition of 10% MACl present the highest PCE of 23.61%, which is much higher than the value (16.72%) for the control device.

## Data Availability

Dataset available on request from the authors.

## Supplementary Materials

The following supporting information can be downloaded at: https://www.mdpi.com/article/10.3390/nano15040292/s1, Figure S1: AFM 3D images of perovskite films; Figure S2: SEM images of perovskites basing on 5%, 20%, and 40% MACl additives; Figure S3: Distribution histograms of perovskite grain sizes basing on different concentrations of MACl additives; Figure S4: AFM images of perovskites basing on 5%, 20%, and 40% MACl additives, and histograms of AFM roughness. Figure S5: XRD of perovskites basing on 5%, 20%, and 40% MACl additives. Figure S6: Absorbance of perovskites basing on 5%, 20%, and 40% MACl additives; Figure S7: PL of perovskites basing on 5%, 20%, and 40% MACl additives; TSCPC of perovskites basing on 0%, 5%, 10%, 20%, and 40% MACl additives; Figure S8: J-V curves of PSCs with 5%, 20%, and 40% MACl additives; Figure S9: Light intensity dependence of Voc of PSCs with 5%, 20%, and 40% MACl additives; Table S1: Average performance of devices basing on different percentages of MACl additives of PSCs; Table S2: Performance comparison table of devices basing on the introduction of MACl additives.

## References

[1] Kojima A., Teshima K., Shirai Y., Miyasaka T. (2009). Organometal Halide Perovskites as Visible-Light Sensitizers for Photovoltaic Cells. J. Am. Chem. Soc..

[2] Gong C., Li H., Xu Z., Li Y., Wang H., Zhuang Q., Wang A., Li Z., Guo Z., Zhang C. (2024). Efficient and stable inverted perovskite solar cells enabled by homogenized PCBM with enhanced electron transport. Nat. Commun..

[3] Yeom K.M., Kim S.U., Woo M.Y., Noh J.H., Im S.H. (2020). Recent Progress in Metal Halide Perovskite-Based Tandem Solar Cells. Adv. Mater..

[4] Liu S., Li J., Xiao W., Chen R., Sun Z., Zhang Y., Lei X., Hu S., Kober-Czerny M., Wang J. (2024). Buried interface molecular hybrid for inverted perovskite solar cells. Nature.

[5] Zhao Y., Cruse K., Abdelsamie M., Ceder G., Sutter-Fella C.M. (2021). Synthetic approaches for thin-film halide double perovskites. Matter.

[6] Li T., Pan Y., Wang Z., Xia Y., Chen Y., Huang W. (2017). Additive engineering for highly efficient organic–inorganic halide perovskite solar cells: Recent advances and perspectives. J. Mater. Chem. A.

[7] Cao X., Zhi L., Jia Y., Li Y., Zhao K., Cui X., Ci L., Zhuang D., Wei J. (2019). A Review of the Role of Solvents in Formation of High-Quality Solution-Processed Perovskite Films. ACS Appl. Mater. Interfaces.

[8] Mateen M., Arain Z., Liu X., Iqbal A., Ren Y., Zhang X., Liu C., Chen Q., Ma S., Ding Y. (2020). Boosting optoelectronic performance of MAPbI3 perovskite solar cells via ethylammonium chloride additive engineering. Sci. China Mater..

[9] Bi L., Fu Q., Zeng Z., Wang Y., Lin F.R., Cheng Y., Yip H.L., Tsang S.W., Jen A.K. (2023). Deciphering the Roles of MA-Based Volatile Additives for alpha-FAPbI(3) to Enable Efficient Inverted Perovskite Solar Cells. J. Am. Chem. Soc..

[10] Huang Z., Bai Y., Huang X., Li J., Wu Y., Chen Y., Li K., Niu X., Li N., Liu G. (2023). Anion-pi interactions suppress phase impurities in FAPbI_3_ solar cells. Nature.

[11] Wang C., He B., Fu M., Su Z., Zhang L., Zhang J., Mei B., Gao X. (2024). Influence of MACl on the Crystallization Kinetics of Perovskite via a Two-Step Method. Crystals.

[12] Shen X., Gallant B.M., Holzhey P., Smith J.A., Elmestekawy K.A., Yuan Z., Rathnayake P., Bernardi S., Dasgupta A., Kasparavicius E. (2023). Chloride-Based Additive Engineering for Efficient and Stable Wide-Bandgap Perovskite Solar Cells. Adv. Mater..

[13] Ding B., Ding Y., Peng J., Romano-deGea J., Frederiksen L.E.K., Kanda H., Syzgantseva O.A., Syzgantseva M.A., Audinot J.N., Bour J. (2024). Dopant-additive synergism enhances perovskite solar modules. Nature.

[14] Kim M., Kim G.-H., Lee T.K., Choi I.W., Choi H.W., Jo Y., Yoon Y.J., Kim J.W., Lee J., Huh D. (2019). Methylammonium Chloride Induces Intermediate Phase Stabilization for Efficient Perovskite Solar Cells. Joule.

[15] Cuzzupè D.T., Öz S.D., Ling J., Illing E., Seewald T., Jose R., Olthof S., Fakharuddin A., Schmidt-Mende L. (2023). Understanding the Methylammonium Chloride-Assisted Crystallization for Improved Performance of Lead-Free Tin Perovskite Solar Cells. Adv. Sol. RRL.

[16] Wu G., Cai M., Cao Y., Li Z., Zhang Z., Yang W., Chen X., Ren D., Mo Y., Yang M. (2022). Enlarging grain sizes for efficient perovskite solar cells by methylamine chloride assisted recrystallization. J. Energy Chem..

[17] Odysseas Kosmatos K., Theofylaktos L., Giannakaki E., Deligiannis D., Konstantakou M., Stergiopoulos T. (2019). Μethylammonium Chloride: A Key Additive for Highly Efficient, Stable, and Up-Scalable Perovskite Solar Cells. Energy Environ. Mater..

[18] Liu H., Sun J., Hu H., Li Y., Hu B., Xu B., Choy W.C.H. (2021). Antioxidation and Energy-Level Alignment for Improving Efficiency and Stability of Hole Transport Layer-Free and Methylammonium-Free Tin-Lead Perovskite Solar Cells. ACS Appl. Mater. Interfaces.

[19] Zhang W., Huang L., Guan H., Zheng W., Li Z., Cui H., Zhou S., Liang J., Li G., Wang T. (2023). Bottom-up modification boosts the performance of narrow-bandgap lead–tin perovskite single-junction and tandem solar cells. Energy Environ. Sci..

[20] Deng X., Qi F., Li F., Wu S., Lin F.R., Zhang Z., Guan Z., Yang Z., Lee C.S., Jen A.K. (2022). Co-assembled Monolayers as Hole-Selective Contact for High-Performance Inverted Perovskite Solar Cells with Optimized Recombination Loss and Long-Term Stability. Angew. Chem. Int. Ed. Engl..

[21] Gao J., Liao C., Guo Y., Zhou D., Zeng Z., Cai C. (2021). The effect of methyl ammonium chloride doping for perovskite solar cells on structure, crystallization and power conversion efficiency. Mod. Phys. Lett. B.

[22] Cheng J., Wang L., Zhou P., Liu D., Chen M., Liang Y., Li W., Hu R., Liang G. (2023). Unraveling Its Intrinsic Role of CH3NH3Cl Doping for Efficient Enhancement of Perovskite Solar Cells from Fine Insight by Ultrafast Charge-Transfer Dynamics. Sol. RRL.

[23] Guo Y., Yuan S., Zhu D., Yu M., Wang H., Lin J., Wang Y., Qin Y., Zhang J., Ai X. (2021). Influence of the MACl additive on grain boundaries, trap-state properties, and charge dynamics in perovskite solar cells. Phys. Chem. Chem. Phys..

[24] Chang J., Feng E., Li H., Ding Y., Long C., Gao Y., Yang Y., Yi C., Zheng Z., Yang J. (2023). Crystallization and Orientation Modulation Enable Highly Efcient Doctor Bladed Perovskite Solar Cells. Nano-Micro Lett..

[25] Amalathas A., Landova L., Hajkova Z., Horak L., Ledinsky M., Holovský J. (2020). Controlled Growth of Large Grains in CH3NH3PbI3 Perovskite Films Mediated by an Intermediate Liquid Phase without an Antisolvent for Efficient Solar Cells. ACS Appl. Energy Mater..

