# Supplementary material for: Optimal Methylammounium Chloride Additive for High-Performance Perovskite Solar Cells
Source: Nanomaterials (Basel). 2025 Feb 14;15(4):292. doi: 10.3390/nano15040292 (PMC11858799; doi:10.3390/nano15040292)
Supplement: Supplementary file 1 [file nanomaterials-15-00292-s001.zip › nanomaterials-3415553-supplementary.pdf]

Supporting Information

Optimal Methylammounium Chloride Additive for High-Performance Perovskite Solar Cells

Qinghua Cao[2,#], Hui Liu[2,#], Jiangping Xing[2,#], Bing'e Li[2], Chuangping Liu[2], Fobao Xie[2], Xiaoli Zhang[2], Weiren Zhao[1,*]

[1]Shanwei-GDUT Collaborative Research Institute for Innovation Industrial Technology, Shangwei 730010, China

[2]School of Physics and Opto-Electronic Engineering, Guangdong Provincial Key Laboratory of Sensing Physics and System Integration Applications, Guangdong University of Technology, Guangzhou 510006, China

[#]Contributing equally to this work

*Corresponding Author E-mail: zwrab@163.com;

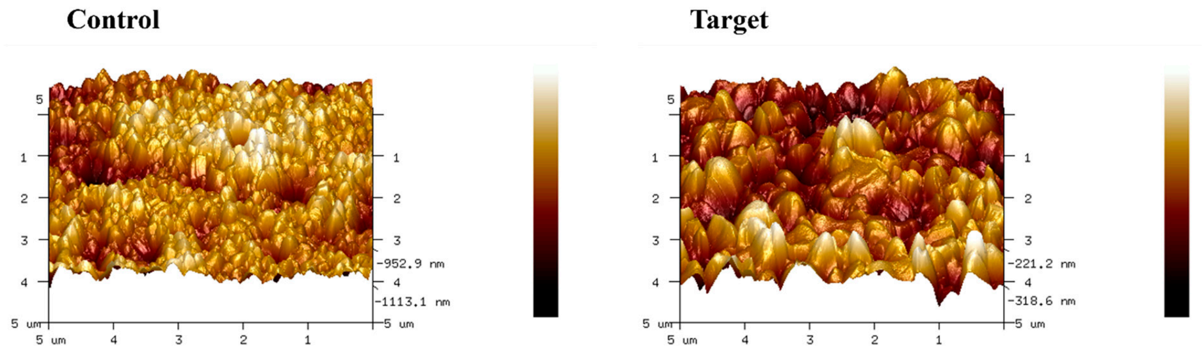

Figure S1. AFM 3D images of perovskite films.

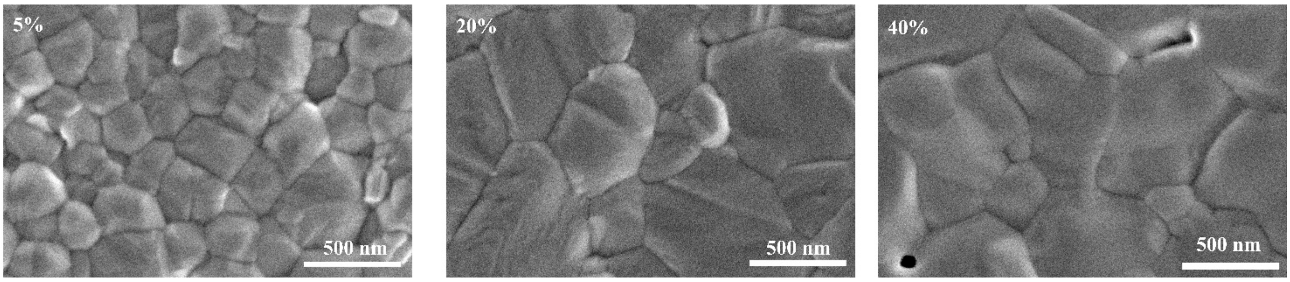

Figure S2. SEM images of perovskites basing on 5%, 20%, and 40% MACl additives.

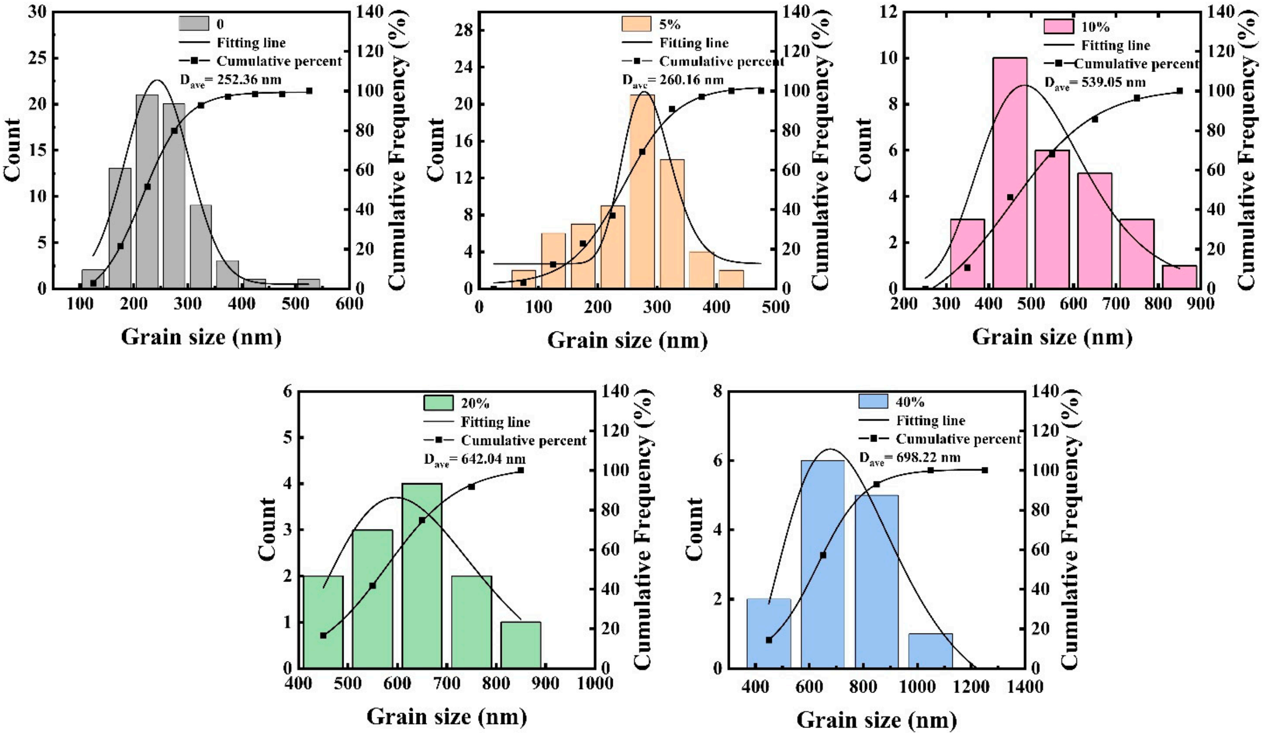

Figure S3. Distribution histograms of perovskite grain sizes basing on different concentrations of MACl additives.

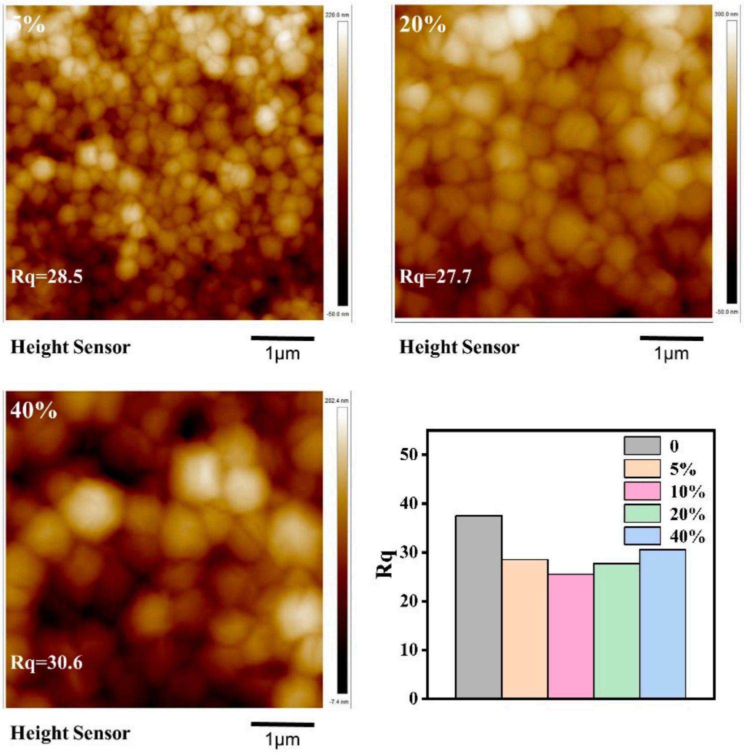

Figure S4. AFM images of perovskites basing on 5%, 20%, and 40% MACl additives, and histograms of AFM roughness.

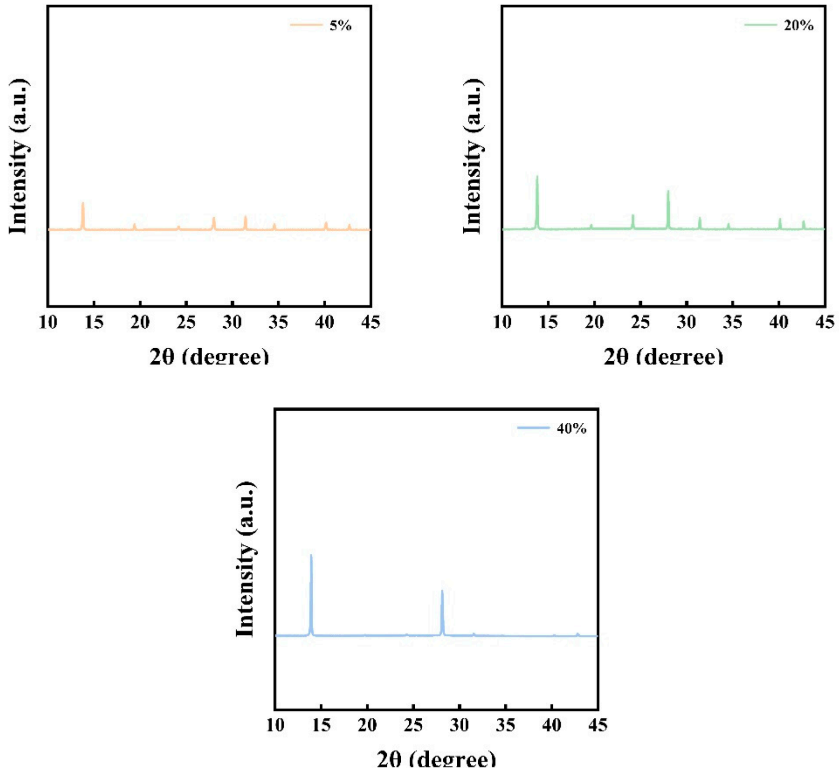

Figure S5. XRD of perovskites basing on 5%, 20%, and 40% MACl additives.

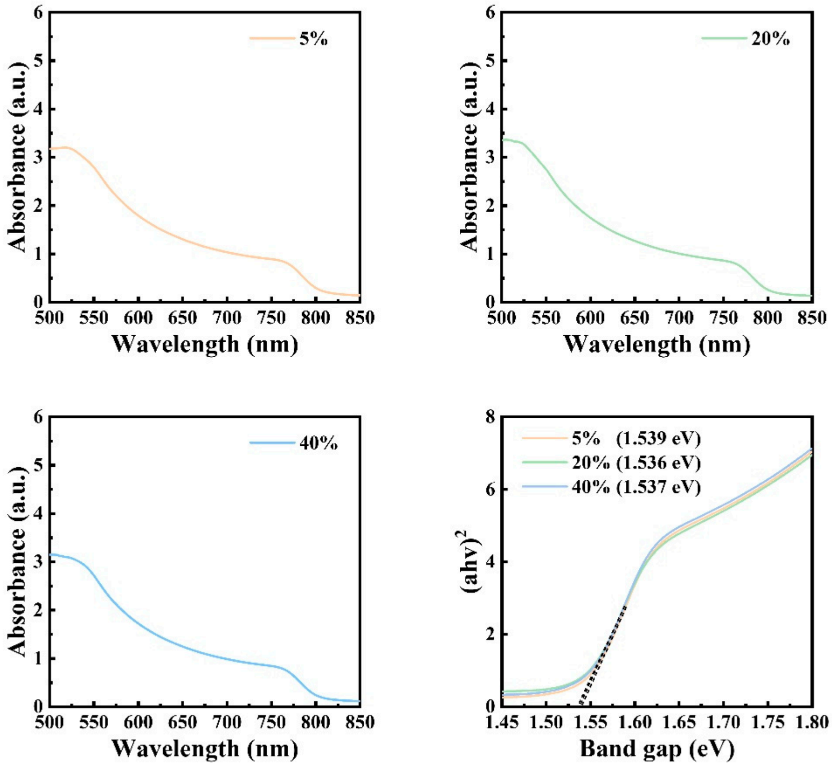

Figure S6. Absorbance of perovskites basing on 5%, 20%, and 40% MACl additives.

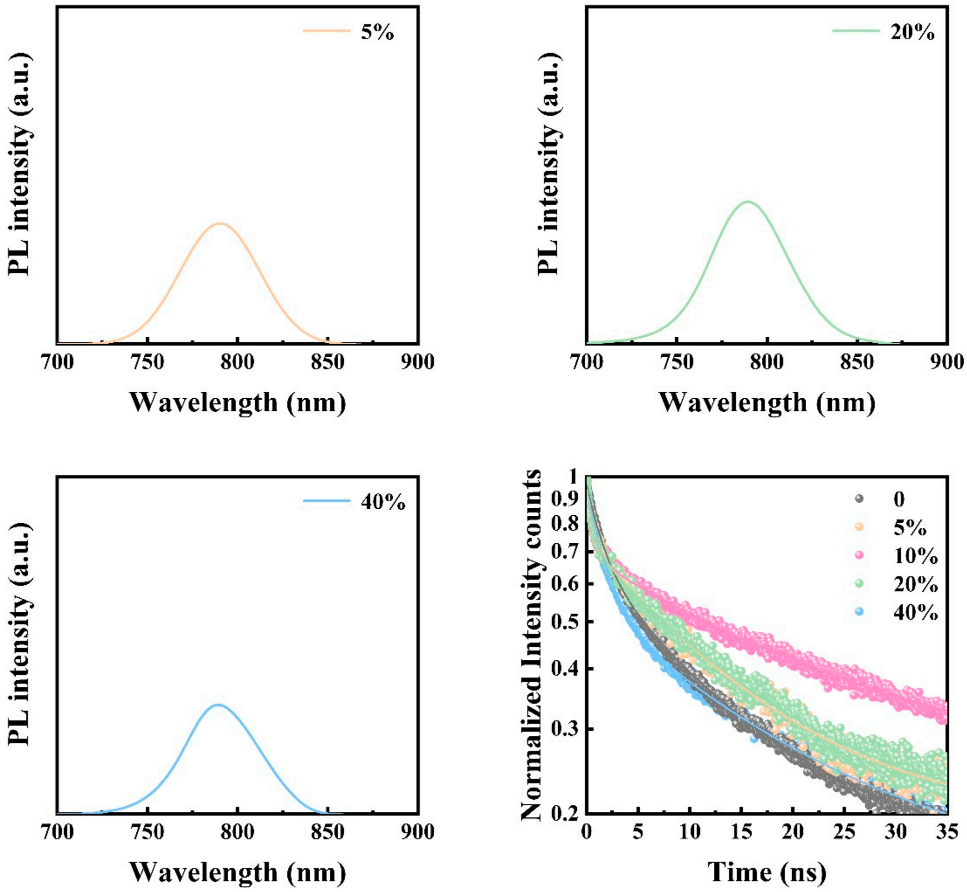

Figure S7. PL of perovskites basing on 5%, 20%, and 40% MACl additives; TSCPC of perovskites basing on 0%, 5%, 10%, 20%, and 40% MACl additives.

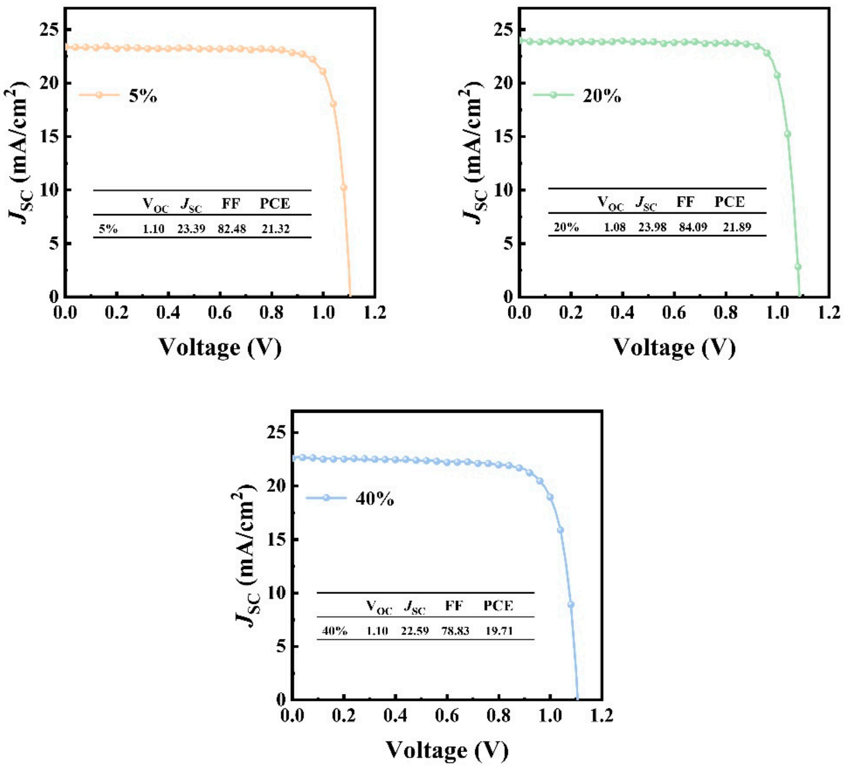

Figure S8. J-V curves of PSCs with 5%, 20%, and 40% MACl additives.

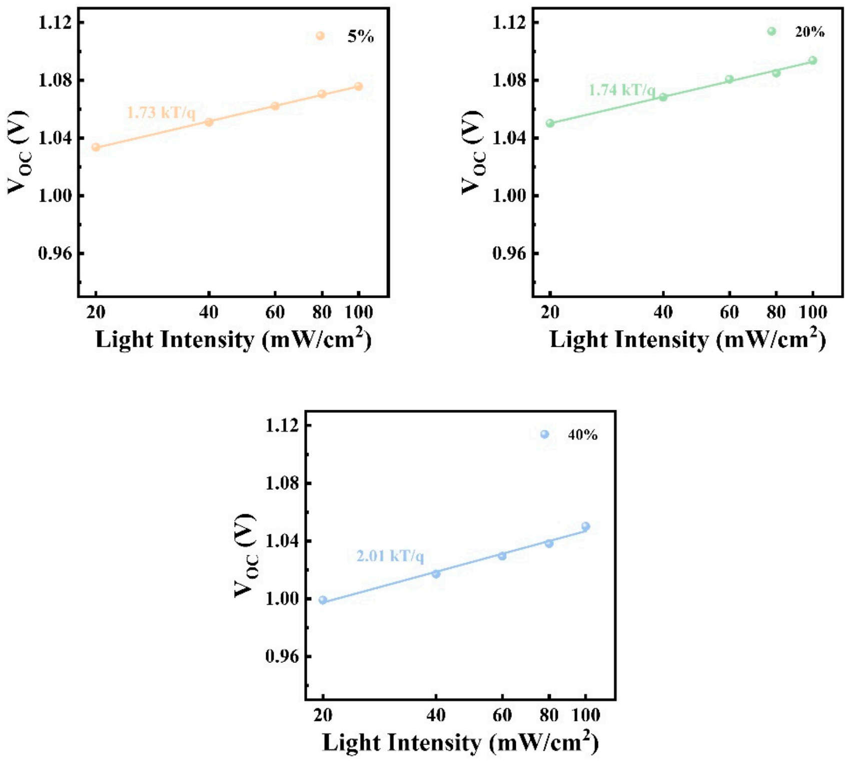

Figure S9. Light intensity dependence of Voc of PSCs with 5%, 20%, and 40% MACl additives.

Table S1. Average performance of devices basing on different percentages of MACl additives of PSCs.

**Percentages**	**PCE (%)**	**V_{OC} (V)**	**FF (%)**	**J_{SC} (mA/cm^2)**
MACl (0 %)	16.41	0.99	78.74	21.07
MACl (5 %)	20.43	1.08	81.81	22.95
**MACl (10 %)**	**22.12**	**1.11**	**82.66**	**23.93**
MACl (20 %)	20.3	1.1	80.2	22.96
MACl (40 %)	17.43	1.05	75.55	21.95

Table S2. Performance comparison table of devices basing on the introduction of MACl additives.

Component	J_{SC} (mA/cm^2)	V_{OC} (V)	FF (%)	PCE (%)	Reference
FTO/c-TiO_2/m-TiO_2)/$(FAPbI_3)_{0.85}(MAPbBr_3)_{0.15}$ (MACl)/Spiro-OMeTAD/Au	23.81	1.11	66.5	17.59	1
FTO/SnO2/$FA_{0.955}MA_{0.045}Pb(I_{0.955}Br_{0.045})_3$(MACl)/Spiro-OMeTAD/Au.	24.66	1.138	84.1	23.60	2
FTO/NiO/$MAPbI_3$(MACl)/PC61BM/BCP/Ag	21.81	1.17	81.94	20.89	3
ITO/SnO_2/$FAPbI_3$(MACl)/Spiro-OMeTAD/Au	24.98	1.14	79.22	22.63	4
FTO/c-TiO_2/m-TiO_2/$FAPbI_3$(MACl)/PEAI//Spiro-OMeTAD/Au	25.92	1.13	82.00	24.02	5
ITO/PTAA/$FAPbI_3$(MACl)/C60/BCP/Ag	25.06	1.12	82.00	23.1	6
FTO/SnO_2/$MAPbI_3$(MACl)/Spiro-OMeTAD/Au.	22.15	1.07	78	18.49	7
ITO/SnO2/FA1-xMAxPb（I1-yBry）3(MACl)/Spiro OMeTAD/Ag	23.41	1.18	81.07	22.45	8
FTO/c-TiO_2/m-TiO_2/$CH_3NH_3PbI_3$(MACl)/spiro-OMeTAD/Au	22.8	1.039	77.7	18.4	9
FTO/MeO-2PACz/Perovskite/PEAI/$PC_{61}BM$/BCP/Ag	24.71	1.14	83.64	23.61	This work

References

1. Gao, J., Liao, C., Guo, Y., Zhou, D., Zeng, Z. & Cai, C. The effect of methyl ammonium chloride doping for perovskite solar cells on structure, crystallization and power conversion efficiency, 2021, 35, 2150096. doi:10.1142/S0217984921500962.

2. Cheng, J., Wang, L., Zhou, P., Liu, D., Chen, M., Liang, Y., Li, W., Hu, R. & Liang, G. Unraveling Its Intrinsic Role of CH3NH3Cl Doping for Efficient Enhancement of Perovskite Solar Cells from Fine Insight by Ultrafast Charge-Transfer Dynamics. Sol. RRL 2023, 7, 2201039, doi: 10.1002/solr.202201039.

3. Wu, G., Cai, M., Cao, Y., Li, Z., Zhang, Z., Yang, W., Chen, X., Ren, D., Mo, Y., Yang, M., Liu, X. & Dai, S. Enlarging grain sizes for efficient perovskite solar cells by methylamine chloride assisted recrystallization. Journal of Energy Chemistry, 2022, 65, 55-61, doi:10.1016/j.jechem.2021.05.026.

4. Wang, C., He, B., Fu, M., Su, Z., Zhang, L., Zhang, J., Mei, B. & Gao, X. Influence of MACl on the Crystallization Kinetics of Perovskite via a Two-Step Method. Crystals,2024, 14, doi:10.3390/cryst14050399.

5. Kim, M., Kim, G.-H., Lee, T. K., Choi, I. W., Choi, H. W., Jo, Y., Yoon, Y. J., Kim, J. W., Lee, J., Huh, D., Lee, H., Kwak, S. K., Kim, J. Y. & Kim, D. S. Methylammonium Chloride Induces Intermediate Phase Stabilization for Efficient Perovskite Solar Cells. Joule,2019, 3, 2179-2192, doi:10.1016/j.joule.2019.06.014.

6. Bi, L., Fu, Q., Zeng, Z., Wang, Y., Lin, F. R., Cheng, Y., Yip, H. L., Tsang, S. W. & Jen, A. K. Deciphering the Roles of MA-Based Volatile Additives for alpha-FAPbI(3) to Enable Efficient Inverted Perovskite Solar Cells. J. Am. Chem. Soc.2023, 145, 5920-5929, doi:10.1021/jacs.2c13566.

7. Guo, Y., Yuan, S., Zhu, D., Yu, M., Wang, H., Lin, J., Wang, Y., Qin, Y., Zhang, J. & Ai, X. Influence of the MACl additive on grain boundaries, trap-state properties, and charge dynamics in perovskite solar cells. Phys. Chem. Chem. Phys., 2021, 23, 6162. doi: 10.1039/d0cp06575g.

8. Chang, J., Feng, E., Li, H., Ding, Y., Long, C., Gao, Y.,Yang, Y., Yi, C., Zheng, Z. & Yang, J. Crystallization and Orientation Modulation Enable Highly Efcient Doctor Bladed Perovskite Solar Cells. Nano-Micro Lett. 2023, 15, 164, doi: 10.1007/s40820-023-01138-x.

9. Amalathas, A., Landova, L., Hajkova, Z., Horak, L. & Ledinsky. Controlled Growth of Large Grains in CH3NH3PbI3 Perovskite Films Mediated by an Intermediate Liquid Phase without an Antisolvent for Efficient Solar Cells. ACS Appl. Energy Mater. 2020, 3, 12484-12493, doi: 10.1021/acsaem.0c02441.
